# A meta-analysis of the effects of transnasal high-flow oxygen therapy in gastrointestinal endoscopy

**DOI:** 10.3389/fmed.2024.1419635

**Published:** 2024-06-27

**Authors:** Chen Wei, Shaoyong Ma, Lili Jiang, Jingwen Wang, Liping Yuan, Yingying Wang

**Affiliations:** ^1^Nursing Department, Yijishan Hospital of Wannan Medical College, Wuhu, Anhui, China; ^2^School of Nursing, Wannan Medical College, Wuhu, Anhui, China; ^3^Department of Emergency, Yijishan Hospital of Wannan Medical College, Wuhu, Anhui, China

**Keywords:** high flow nasal cannula, gastrointestinal endoscopy, gastroenteroscopy, hypoxemia, meta-analysis

## Abstract

**Purpose:**

This study aimed to systematically evaluate the clinical effects of using transnasal high-flow nasal cannula (HFNC) and conventional oxygen therapy (COT) in patients undergoing gastrointestinal endoscopy.

**Methods:**

A comprehensive literature search was conducted from 2004 to April 2024 to collect relevant studies on the application of HFNC in patients undergoing gastrointestinal endoscopy. Multiple Chinese and English databases, including China National Knowledge Infrastructure (CNKI), Wanfang Data, Web of Science, PubMed, and Cochrane Library, were searched systematically for randomized controlled trials (RCTs). Two researchers independently screened the literature, extracted data, and assessed the risk of bias in the included studies. RevMan 5.4 software was utilized for conducting the network meta-analysis.

**Results:**

A total of 12 RCTs involving 3,726 patients were included. Meta-analysis results showed that HFNC reduced the incidence of hypoxemia and improved the minimum oxygen saturation (SpO_2_) compared with COT [odds ratio (OR) = 0.39, 95% confidence interval (CI): 0.29–0.53], [mean difference (MD) = 4.07, 95% CI: 3.14–5.01], and the difference was statistically significant. However, the baseline SpO_2_ levels and incidence of hypercapnia were not statistically significantly different between the HFNC and COT groups [MD = −0.21, 95% CI: −0.49–0.07]; [OR = 1.43, 95% CI: 0.95–2.15]. In terms of procedure time, the difference between HFNC and COT was not statistically significant, and subgroup analyses were performed for the different types of studies, with standard deviation in the gastroscopy group (MD = 0.09, 95% CI: −0.07–0.24) and the endoscopic retrograde cholangiopancreatography group (MD = 0.36, 95% CI: −0.50–1.23). The results demonstrated a significant reduction in the adoption of airway interventions in the HFNC group compared to the COT group (OR = 0.16, 95% CI: 0.05–0.53), with a statistically significant difference; this result was consistent with those of the included studies.

**Conclusion:**

The application of HFNC improves the incidence of hypoxemia, enhances oxygenation, and reduces airway interventions during gastrointestinal endoscopy. However, HFNC does not significantly affect baseline SpO_2_, hypercapnia, or procedure time. The limitations of this study must be acknowledged, and further high-quality studies should be conducted to validate these findings.

## 1 Introduction

Significant advancements in medical technology and the increasing need for comprehensive patient care have made gastrointestinal endoscopy a vital clinical modality for diagnosing and treating various gastrointestinal disorders. This minimally invasive procedure boasts notable advantages, including rapid recovery time and shorter duration. Gastrointestinal endoscopy includes a range of procedures such as gastroscopy, colonoscopy, and retrograde cholangiopancreatography. It involves the insertion of an endoscope through the oral cavity, precise navigation of the endoscope into the esophagus or stomach, and visualization of the internal anatomy using a camera system, enabling the timely and accurate identification of lesions (1). Considering patient discomfort, the majority of gastrointestinal endoscopies are conducted under sedation (2). Clinical guidelines strongly recommend routine sedation during endoscopic procedures due to its effectiveness in alleviating patient anxiety, improving compliance, and enhancing patient acceptance and adherence (3). However, sedation usage carries potential complications, including hypoxemia, hypercarbia, hypotension, and asphyxia, with hypoxemia being the most frequently encountered during endoscopy. Hypoxemia occurs in 3%−30% of gastrointestinal endoscopy cases, making it one of the most prevalent complications (4–6). In severe cases, immediate airway interventions such as mandibular support, nasal airway insertion, mask ventilation, and endotracheal intubation are necessary. Therefore, developing an appropriate oxygenation strategy for sedated patients is of utmost importance.

Conventional oxygen therapy (COT) often falls short in meeting the oxygenation requirements of sedated patients because of the limited oxygen flow used in this method. By contrast, high-flow nasal cannula (HFNC) is a novel non-invasive high-flow oxygen therapy technique. This technique uses an unsealed HFNC to provide a continuous supply of oxygen while ensuring stable temperature, humidity, and a consistent concentration of inhaled oxygen throughout the therapy (7, 8). HFNC has demonstrated its effectiveness in managing critically ill patients (9, 10) and has found application in various procedures, including bronchial endoscopy. Nevertheless, the specific benefits of HFNC in the context of gastrointestinal endoscopy remain largely unexplored, and a comprehensive systematic evaluation in this domain is currently lacking. To fully understand the potential advantages and clinical implications of using HFNC in gastrointestinal endoscopy, further research endeavors are warranted. Recent studies conducted by Kim et al. and Nay et al. (11, 12) have revealed a noteworthy reduction in the incidence of hypoxemia and improvement in minimum SpO_2_ levels with the implementation of HFNC. Conversely, Sawase et al. (13) did not observe a significant difference in the occurrence of hypoxemia between the two groups. In addition, in one of the aforementioned studies (11), the HFNC group demonstrated avoidance of any airway interventions during endoscopy. These findings highlight the potential of HFNC in improving oxygenation and minimizing the need for airway interventions during gastrointestinal endoscopy procedures. It is noteworthy that high-concentration oxygen therapy carries the potential risk of inducing lung atelectasis and decreasing the level of alveolar surface active substances. These effects can adversely impact pulmonary ventilation and contribute to the development of lung injury (14, 15). Furthermore, the incidence of hypercapnia or the duration of gastrointestinal endoscopy did not show improvements. Currently, HFNC is rapidly advancing and presents numerous advantages. However, the majority of studies on oxygen therapy during gastrointestinal endoscopy, both nationally and internationally, have been conducted in single-center settings with limited sample sizes. This has led to inconsistent outcome indicators and a lack of comprehensive systematic research and evaluation. Two meta-analyses have investigated the impact of implementing HFNC on patients undergoing gastrointestinal endoscopy. Lee et al.'s meta-analysis could not provide extensive insights into the heterogeneity in the results because of the small number of randomized controlled trials (RCTs) in the study (16). Similarly, Tao et al.'s meta-analysis showed high heterogeneity, which indicates that HFNC may not effectively reduce the overall incidence of hypoxemia during gastrointestinal endoscopic procedures (17). Given the variability in the findings of these two studies, more recent and relevant studies have been assessed to supplement these findings (16, 17). Therefore, this study aims to conduct a meta-analysis to objectively assess whether HFNC can effectively reduce the incidence of hypoxemia and the need for airway interventions during gastrointestinal endoscopy. This objective can be achieved through a comprehensive literature search, review, and quality evaluation. The findings of this study are expected to provide a robust theoretical foundation for the adoption of HFNC in clinical practice for patients undergoing gastrointestinal endoscopy.

## 2 Materials and methods

### 2.1 Literature search

A comprehensive literature search was conducted across various databases, including China National Knowledge Infrastructure (CNKI), Wanfang Data, Web of Science, PubMed, and Cochrane Library. The primary objective of this search was to identify RCTs that investigated the use of HFNC in patients undergoing gastrointestinal endoscopy from 2004 to April 2024. As the search strategy, we employed a combination of subject terms and keywords, including terms such as “high flow nasal cannula,” “HFNC,” “high flow oxygen therapy,” “HFNO,” “high flow nasal prong,” and “high flow nasal oxygenation.” These terms were cross-referenced with terms related to procedures, such as “EGD,” “endoscope,” “ERCP,” “esophagogastroduodenoscopy,” “endoscopy,” and “endoscopic retrograde cholangiopancreatography.” The search was limited to English and Chinese literature to ensure a comprehensive review of the relevant studies.

### 2.2 Inclusion and exclusion criteria

The inclusion criteria for this study were as follows: (1) Study population (P): patients undergoing gastrointestinal endoscopy; (2) Age ≥ 18 years; (3) Intervention method (I): high-flow nasal cannula; Comparison (C): conventional oxygen therapy (COT) (intervention method: comparison of the effectiveness of HNFC vs. COT in patients undergoing gastrointestinal endoscopy, with the HFNC group receiving oxygen flow rates ranging 20–60 L/min and the COT group receiving oxygen flow rates ranging 1–8 L/min predominantly through naso-oxygenated tubing); (4) Outcome indicators (O): incidence of hypoxemia, baseline SpO_2_, minimum SpO_2_, hypercapnia, incidence of airway interventions (e.g., chin lift, mandibular push-up, or nasopharyngeal airway insertion), and procedure time; and (5) Study design (S): RCT.

The exclusion criteria were as follows: (1) Literature in reviews, conferences, case reports, etc.; (2) incomplete extracted data; and (3) unavailability of full text.

### 2.3 Data extraction

Two independent reviewers meticulously conducted a comprehensive literature screening to extract relevant data. In the event of any discrepancies, both reviewers engaged in discussions or sought the input of a third party for resolution. The literature screening process strictly adhered to predefined inclusion and exclusion criteria to ensure consistency and rigor. The extracted data included essential details such as article author, title, publication date, intervention specifics, sample size, various outcome indicators, and an assessment of the risk of bias. Furthermore, to enhance the reliability of the extracted data, outcome measures from both groups were cross-checked for accuracy. By implementing these rigorous screening and data extraction procedures, we aimed to minimize bias and ensure the reliability of the data used in our study analysis.

### 2.4 Quality evaluation

The quality assessment of the included literature was conducted independently by two trained personnel. In the event of any disagreement regarding the evaluation results, discrepancies were resolved through discussions between the two personnel or with the assistance of a third-party expert. The quality of the included studies was evaluated using the bias evaluation tool outlined in the Cochrane Handbook for Systematic Reviews of Interventions 5.2.0. The assessment encompassed seven key items: random sequence generation (selective bias), allocation concealment (selective bias), blinding of implementers and participants (implementation bias), blinding of outcome evaluators (observation bias), integrity of results (follow-up bias), selective reporting of study results (reporting bias), and other potential sources of bias. The evaluation results were categorized as “low risk of bias,” “unclear,” or “high risk of bias” based on the assessment criteria.

### 2.5 Statistical analysis

The meta-analysis was conducted using Cochrane Review Manager 5.4 software. The Mantel–Haenszel method was employed for categorical outcomes, and the pooled results were expressed as odds ratios (OR) with corresponding 95% confidence intervals (CI). Meanwhile, the inverse variance method was utilized for continuous outcomes, and the effect measures were presented as mean differences (MD) or standardized mean differences (SMD) with 95% CIs. Statistical heterogeneity was assessed using the *I*^2^ statistic, with substantial heterogeneity defined as an *I*^2^ value >50%. Sensitivity analysis was performed using a leave-one-out approach to evaluate the potential impact of individual trials on the overall meta-analytical results. All analyses were two-sided, and statistical significance was defined as a *p*-value of < 0.05.

## 3 Results

### 3.1 Literature screening and results

**Figure 1:**
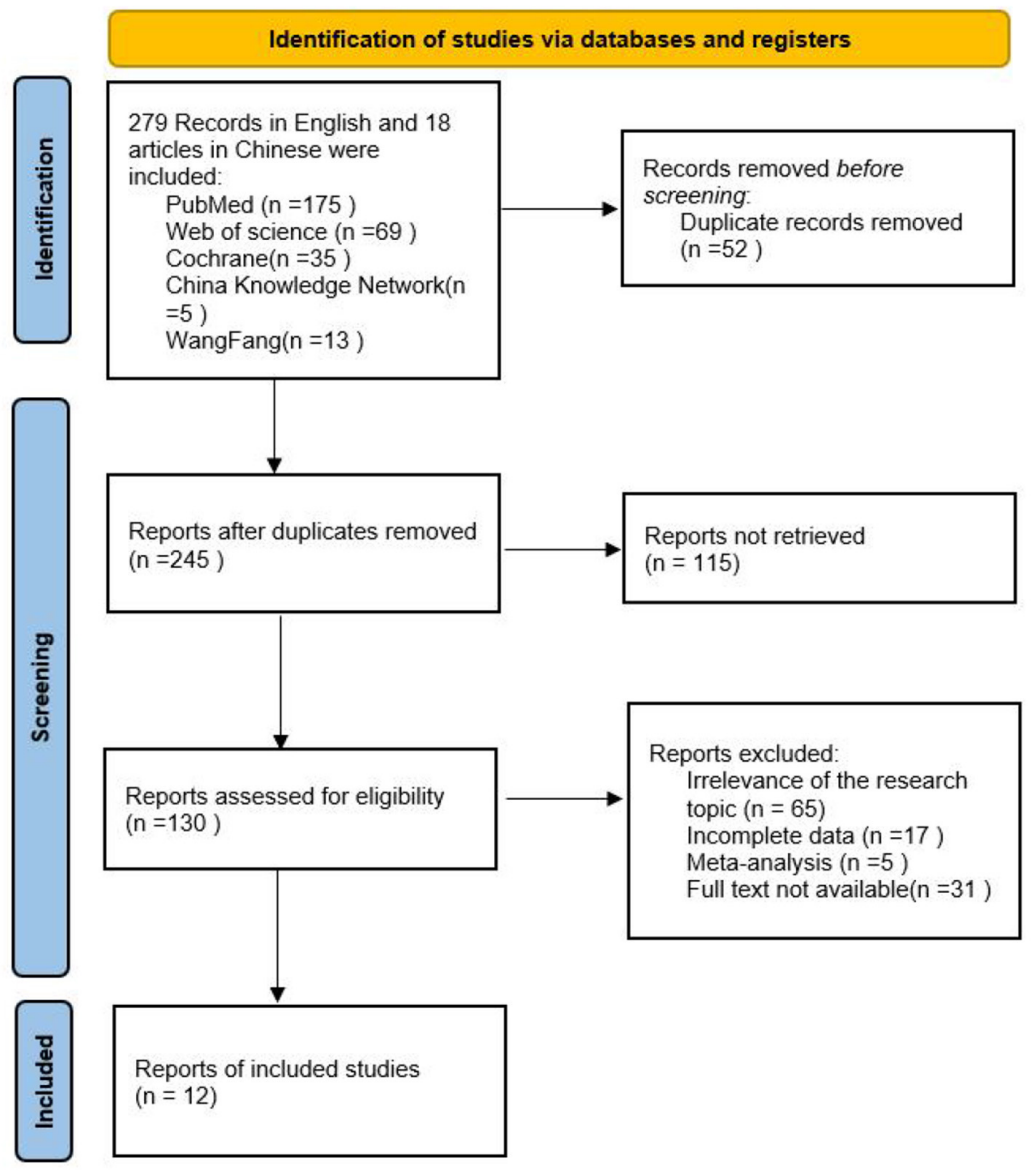
Basic characteristics of articles.

A comprehensive search was conducted across multiple databases, including CNKI, Wanfang Data, Web of Science, PubMed, and Cochrane Library, resulting in an initial retrieval of 297 articles. After removing duplicates, a total of 245 articles remained. Subsequently, 115 articles were excluded based on the evaluation of titles and abstracts. Finally, 12 articles (11–13, 18–26) were selected for inclusion after a thorough examination of the full-text publications. The detailed screening process is shown in Figure 1.

### 3.2 Basic characteristics of the included articles

A total of 12 RCTs, involving 3,726 patients, were included in the analysis. The efficacy of HFNC compared to COT was assessed in patients undergoing gastrointestinal endoscopy. The HFNC group received oxygen flow rates of 20–60 L/min, whereas the COT group received oxygen flow rates of 1–8 L/min predominantly through nasal oxygen tubing. The basic characteristics of the included studies and the patients therein are presented in Table 1.

**Table 1:** Basic characteristics of articles.

	**Quality assessment**						
**References**	**Country**	**Sample size (T/C)**	**Mean age (years)**		**Intervention**		**Outcome**
			**HFNC**	**COT**	**HFNC**	**COT**	
Kim et al. (11)	Korea	36/36	67.3 ± 14.4	65.3 ± 13.41	100% oxygen 50 L/min	100% oxygen 5 L/min	①②③⑤⑥
Thiruvenkatarajan et al. (25)	Australia	65/66	69.1 ± 17.7	65.5 ± 18.9	30–50 L/min	Oxygen 4 L/min	①③④⑥
Nay et al. (12)	France	191/188	62.9 ± 12.7	63.3 ± 12.0	40 L/min	Standard Oxygen Flow Rate	①②⑤
Lin et al. (21)	China	994/1000	47 ± 18.84	48 ± 18.86	30–60 L/min	2 L/min	①⑤⑥
Riccio et al. (23)	USA	28/31	54 ± 8	59 ± 7	60 L/min	4 L/min	①③⑤⑥
Sawase et al. (13)	Japan	37/38	65.3 ± 30.8	62.6 ± 37.0	40–60 L/min	1–2 L/min	①④
Mazzeffi et al. (22)	USA	132/130	62 ± 15	62 ± 13	20 L/min	6 L/min	①②④⑥
Zhang et al. (26)	China	123/123	69.6 ± 3.8	70 ± 3.0	30 L/min	8 L/min	①③⑤⑥
Teng et al. (24)	China	50/51	46.65 ± 15.37	51.56 ± 12.52	30 L/min	5 L/min	⑤⑥
Lee et al. (20)	Korea	95/92	78 ± 7	79 ± 7	50 L/min	5 L/min	①⑤
Qiu et al. (19)	China	52/52	76.75 ± 4.59	76.27 ± 3.95	30 L/min	5 L/min	③⑤
Chen et al. (18)	China	58/58	81.4 ± 6.7	79.8 ± 6.4	30–40 L/min	3–5 L/min	①②⑤⑥

HFNC, High-flow nasal cannula; COT, Conventional oxygen therapy; T, Test group; C, Control group; ① Incidence of hypoxemia; ② Baseline SpO_2_; ③ Lowest SpO_2_; ④ Incidence of hypercapnia; ⑤ Duration of surgery; ⑥ Airway interventions.

### 3.3 Evaluation of risk of bias of the included articles

**Figure 2:**
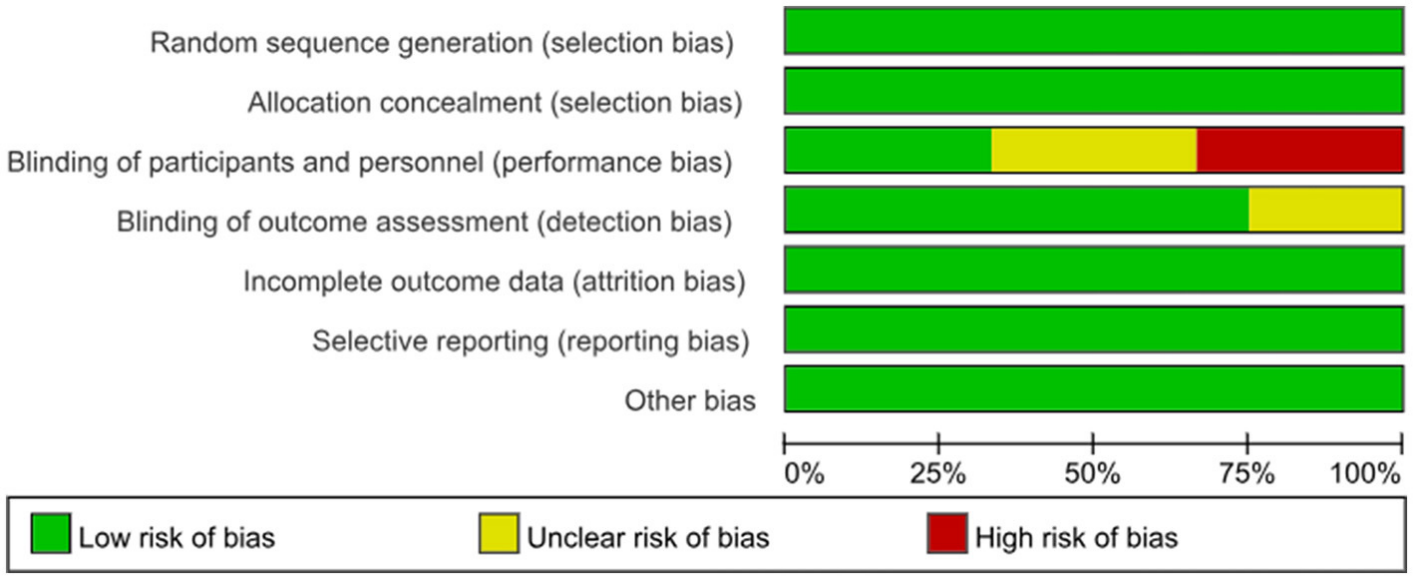
Graph for risk of bias.

The 12 included studies provided detailed descriptions of the methods used for generating random sequences, which were assessed as low risk in terms of bias. The methods primarily employed include a random number table and a computer-generated random number table. A summary of the risk of bias is presented in Figure 2.

### 3.4 Meta-analysis

#### 3.4.1 Incidence of hypoxemia

**Figure 3:**
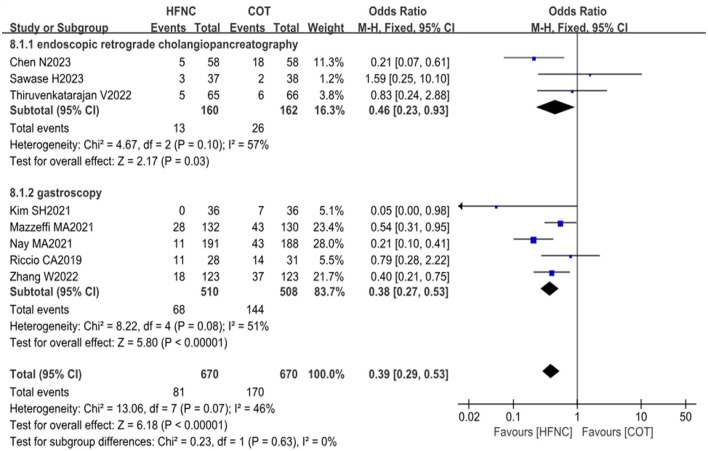
Forest plot comparing the incidence of hypoxemia in the HFNC vs. control groups.

The analysis included a total of eight studies involving 1,340 patients (HFNC group, *n* = 670; control group, *n* = 670) for assessing the impact of HFNC on the incidence of hypoxemia. In most of the included literature, hypoxemia was defined as SpO_2_ < 90%. (11, 13, 25) Subgroup analyses were performed based on the type of endoscopy. Three studies focused on gastroscopy, while five studies examined endoscopic retrograde cholangiopancreatography. The results of our analysis revealed a significantly lower risk of hypoxemia in the HFNC group compared to the control group for both gastroscopy (OR = 0.46, 95% CI: 0.23–0.93, *P* = 0.03) and endoscopic retrograde cholangiopancreatography (OR = 0.38, 95% CI: 0.27–0.53, *P* < 0.00001). These findings were consistent across different examination methods, as depicted in Figure 3.

#### 3.4.2 Baseline SpO_2_

**Figure 4:**
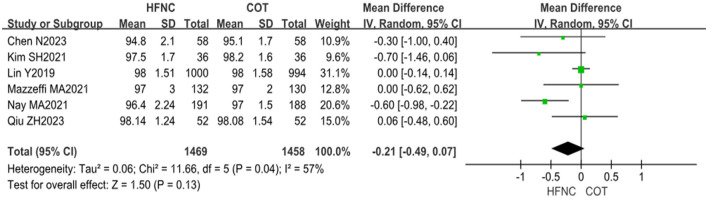
Forest plot comparing baseline SpO_2_ during the procedure in HFNC vs. COT.

Baseline SpO_2_ data from six RCTs involving a total of 2,927 patients were analyzed, with 1,469 patients in the HFNC group and 1,458 patients in the COT group. Heterogeneity testing of the study data yielded a statistically significant result (*P* = 0.04, *I*^2^ = 57%). The meta-analysis indicated no statistically significant difference in baseline SpO_2_ between the two groups during gastrointestinal endoscopy (MD = −0.21, 95% CI: −0.49–0.07, *P* = 0.13), as illustrated in Figure 4.

#### 3.4.3 Lowest SpO_2_

**Figure 5:**
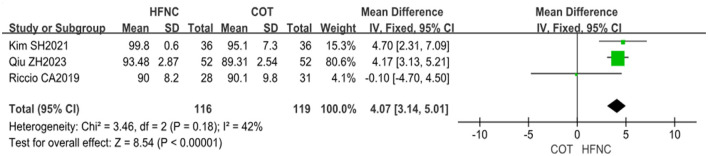
Forest plot comparing the lowest SpO_2_ during the procedure in HFNC vs. COT.

Four studies reported the lowest SpO_2_ of patients recorded during surgery; however, one of the studies (13) utilized a different intervention method under COT and was thus excluded from the analysis. The final analysis included a total of 235 patients, with 116 patients in the HFNC group and 119 patients in the COT group. Heterogeneity testing was conducted on the study data, and non-significant results (*P* < 0.18, *I*^2^ = 42%) were yielded, indicating homogeneity between the two groups. Consequently, the fixed-effect model was selected for the analysis. The meta-analysis revealed a statistically significant difference, with the lowest SpO_2_ in the HFNC group being higher than that in the COT group (MD = 4.07, 95% CI: 3.14–5.01, *P* < 0.00001), as demonstrated in Figure 5.

#### 3.4.4 Incidence of hypercapnia

**Figure 6:**
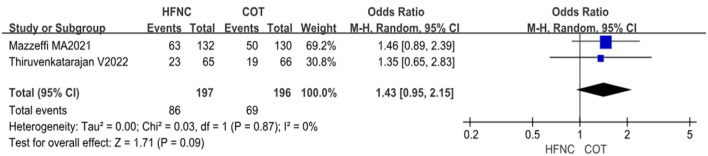
Forest plot comparing the incidence of hypercapnia during the procedure in HFNC vs. COT.

Three studies reported the incidence of hypercapnia; however, the study by Sawase et al. (13) was excluded due to differing criteria for determining hypercapnia compared to the other two studies. The analysis included a total of 393 patients, with 197 in the HFNC group and 196 in the COT group. Heterogeneity testing of the study data demonstrated low heterogeneity (*P* = 0.87, *I*^2^ = 0%), indicating the application of fixed effects for the meta-analysis. The results revealed that the incidence of hypercapnia was slightly higher in the HFNC group compared to the COT group (OR = 1.43, 95% CI: 0.95–2.15, *P* = 0.09), although the difference was not statistically significant (Figure 6).

#### 3.4.5 Duration of surgery

**Figure 7:**
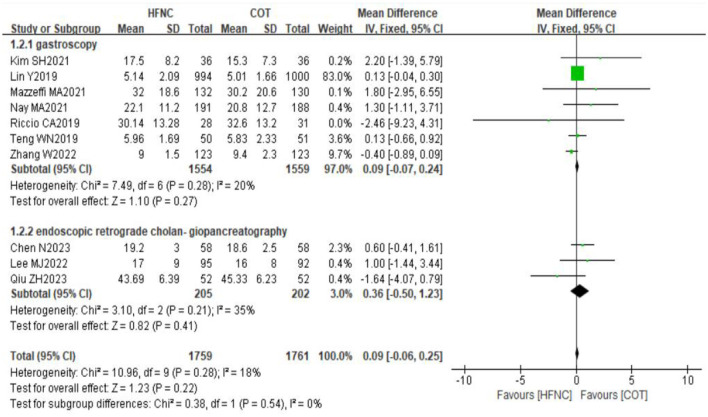
Forest plot comparing the duration of surgery in HFNC vs. COT.

Ten studies reported the duration of surgery in a total of 3,520 patients, with 1,759 in the HFNC group and 1,761 in the COT group. Heterogeneity testing of the study data showed homogeneity between the two groups (*P* = 0.28, *I*^2^ = 18%), allowing for the utilization of a fixed-effect model for the meta-analysis. The results of the meta-analysis indicated no statistically significant difference in the duration of surgery between the HFNC and COT groups (MD = 0.09, 95% CI: −0.06–0.25, *P* = 0.22). Subgroup analysis based on the type of endoscopy (gastroscopy and endoscopic retrograde cholangiopancreatography) also revealed no statistically significant differences between the two groups (Figure 7).

#### 3.4.6 Airway interventions

**Figure 8:**
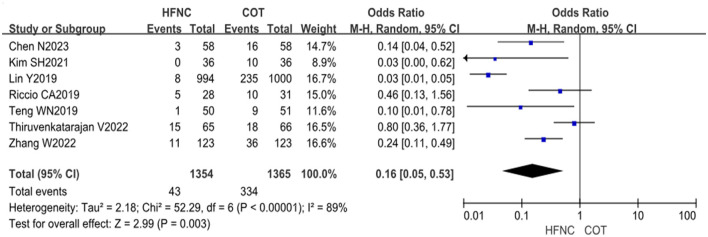
Forest plot comparing the airway interventions during the procedure in HFNC vs. COT.

A total of seven studies, involving 2,719 patients (1,354 in the HFNC group and 1,365 in the COT group), reported the number of airway interventions. Heterogeneity testing of the data revealed significant heterogeneity (*P* < 0.00001, *I*^2^ = 89%). Due to the observed heterogeneity, a random effects model was selected for the analysis. The results demonstrated a significant reduction in the adoption of airway interventions in the HFNC group compared to the COT group (OR = 0.16, 95% CI: 0.05–0.53, *P* = 0.003), indicating a statistically significant difference (Figure 8).

### 3.5 Publication bias

**Figure 9:**
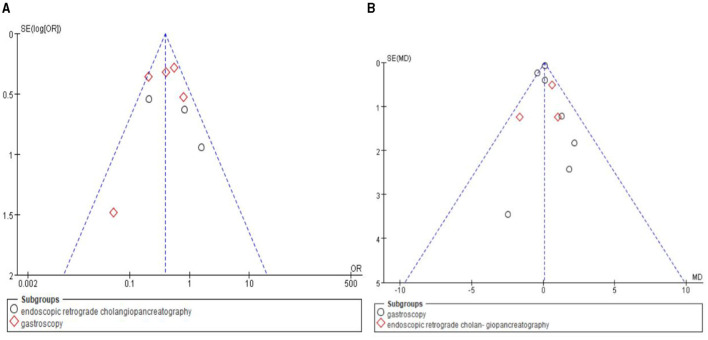
Funnel plot for publication bias. **(A)** Funnel plot of the incidence of hypoxaemia. **(B)** Funnel plot of the duration of surgery.

In this study, funnel plots were used to assess publication bias for the incidence of hypoxemia and procedure time. The results indicated no noticeable publication bias, as demonstrated by the symmetrical and well-distributed nature of the points within the funnel plots. All points fell within the expected range, indicating a low likelihood of significant publication bias. Funnel Figures 9A, and 9B provide an overall symmetrical representation, further supporting this conclusion.

## 4 Discussion

Painless gastrointestinal endoscopy is currently widely recognized as the preferred approach for patients with digestive disorders as it offers improved patient comfort and shorter procedure duration (27). Concurrently, sedation during gastrointestinal examinations further enhances patient satisfaction. In line with these benefits, our study findings demonstrate a significant reduction in the incidence of hypoxemia among patients undergoing gastrointestinal endoscopy when HFNC is used compared to when COT is used. Notably, HFNC maintains a relatively high minimum SpO_2_ level throughout the examination, mitigating the need for airway interventions in the event of complications. These results highlight the potential of HFNC to optimize respiratory support and enhance patient safety during gastrointestinal endoscopic procedures. However, no significant differences were observed in baseline SpO_2_ levels, procedure duration, or the incidence of hypercapnia between the HFNC and control groups. Particularly, a previous study (28) has highlighted the potential risks associated with sedation during gastrointestinal endoscopy, including respiratory depression and hypoxemia. By contrast, our findings support the beneficial effect of HFNC in reducing the incidence of hypoxemia during the procedure. However, it is important to acknowledge the limited availability of literature on the implementation of HFNC in gastrointestinal endoscopy, characterized by a scarcity of studies, small sample sizes, and overall variability in study quality. These shortcomings necessitate further investigation. In addition, comprehensive research is needed to elucidate the indications and contraindications of HFNC in gastrointestinal endoscopy. To advance the field, future studies should prioritize high-quality, multicenter investigations to comprehensively evaluate the effectiveness and safety of HFNC in gastrointestinal endoscopy settings, providing robust evidence for its optimal utilization.

Hypoxemia, characterized by a reduction in arterial oxygen levels, represents a frequently encountered complication during gastrointestinal endoscopy, with reported incidences ranging from approximately 3% to 30% (4–6). To evaluate the efficacy of HFNC in mitigating this risk, our study reviewed eight RCTs. The collective evidence consistently demonstrated a significantly lower incidence of hypoxemia in patients treated with HFNC compared to those receiving COT. Furthermore, a subgroup analysis based on the type of examination revealed remarkable benefits of HFNC in reducing the occurrence of hypoxemia during both gastroscopy and endoscopic retrograde cholangiopancreatography procedures, with no statistically significant difference observed between the two examination types. These findings highlight the potential of HFNC as a valuable respiratory support modality for enhancing patient safety during gastrointestinal endoscopy. Studies by Nay et al. (12) and Zhang et al. (26) provided compelling evidence of the significant improvement in hypoxemia observed in the HFNC group when utilizing an oxygen flow rate of 30 L/min. Sawase et al. (13) performed endoscopic retrograde cholangiopancreatography procedures with a flow rate of 40–60 L/min in the HFNC group and with that of 1–2 L/min in the COT group. Notably, the HFNC group received air rather than supplemental oxygen during the examination to investigate its potential benefits in improving gas exchange. In cases where the patient's SpO_2_ decreased below 90%, oxygen concentration could be added to the HFNC group as necessary to ensure patient safety. The results indicated a higher incidence of hypoxemia in the HFNC group compared to the COT group, although the difference was not statistically significant due to the limited sample size. It is important to note that, in this study, no additional oxygen supplementation was provided to the HFNC group during the examination process, which may have influenced the outcomes; therefore, further verification through additional research is required. However, the occurrence of severe hypoxemia during endoscopy poses a significant challenge as it can interrupt the procedure, necessitating immediate airway interventions, such as jaw lifting, mask ventilation, or invasive ventilation. While this study provided valuable insights into the positive impact of HFNC in gastrointestinal endoscopy, additional clinical research is warranted to validate and further elucidate the effectiveness of this intervention. The findings from such research will offer a more comprehensive understanding of the role of HFNC in optimizing respiratory support, minimizing complications, and improving patient outcomes during gastrointestinal endoscopic procedures.

Furthermore, our study revealed a significant correlation between the utilization of HFNC and a decreased frequency of airway interventions during gastrointestinal endoscopy compared to when COT is used. Importantly, this reduction in airway interventions is associated with a decreased risk of procedural interruption. This study provides strong evidence supporting the efficacy of HFNC in reducing the need for airway interventions during gastrointestinal endoscopy compared to that required when COT is used. One study (11) demonstrated that the use of HFNC eliminated the need for any airway interventions, whereas another large study (21) involving approximately 2,000 participants revealed a staggering 30-fold higher occurrence of airway interventions in the COT group compared to the HFNC group. Specifically, only eight patients in the HFNC group required jaw lifting to optimize ventilation, whereas in the COT group, 235 patients required jaw lifting, 82 patients needed increased oxygen flow, and an additional 2 patients had to switch to face mask ventilation to address hypoxia, potentially leading to procedure discontinuation. In contrast to COT, which often fails to meet the oxygen requirements of patients in clinical practice due to insufficient flow rates, HFNC is a novel method of oxygen therapy that delivers high-flow gases mixed with air-oxygen directly into the patient's nasal cavity, ensuring a consistently higher and stable oxygen flow rate. HFNC also offers several advantages over COT, including the maintenance of warm and moist incoming gas (29, 30), efficient carbon dioxide expulsion from the nasopharyngeal cavity, decreased nasopharyngeal resistance, improved gas entry into the lower airway, and the generation of positive airway pressure, thereby increasing end-expiratory lung volume (10). The presence of severe hypoxemia during endoscopy can lead to procedural interruptions and necessitate immediate airway interventions such as jaw lifting, mask ventilation, or invasive ventilation. This study provides compelling evidence regarding the positive significance of HFNC in the context of gastrointestinal endoscopy, emphasizing the need for future clinical research to validate its effectiveness.

Riccio et al. (23) revealed lower minimum SpO_2_ levels in the HFNC group compared to the COT group specifically among morbidly obese patients (body mass index, BMI ≥ 40 kg/m^2^). Although the planned inspired oxygen concentration (FiO_2_) was initially set to 36%−40% for both the HFNC and COT groups, accurately confirming the actual FiO_2_ in practice proved to be challenging for both groups. The FiO_2_ levels in the COT group could have been higher than those in the HFNC group, potentially affected by patient-related factors. This discrepancy in FiO_2_ levels may explain why no significant difference was observed between the two groups. However, when this particular study was excluded, it became apparent that the minimum SpO_2_ levels were higher in the HFNC group compared to the COT group.

Another study (11) observed that, during endoscopic retrograde cholangiopancreatography performed in the prone position, the COT group exhibited SpO_2_ levels below 90%, whereas the HFNC group maintained higher SpO_2_ levels. The findings of this meta-analysis are consistent with the results reported by Lee et al. (31), providing further evidence that HFNC is effective in maintaining minimum SpO_2_ levels among patients. HFNC, with its high airflow, facilitates dead space lavage, thereby enhancing oxygenation and promoting the clearance of carbon dioxide during sedation (32). Considering the relatively small sample size of the included studies and the need to synthesize findings from meta-analysis and other relevant studies, further investigation is required to understand the role of HFNC in oxygenation management, especially in scenarios where maintaining patient oxygenation stability is critical.

This meta-analysis found no statistically significant difference (*P* > 0.05) in the incidence of hypercapnia between HFNC and COT, which is consistent with the results of a study comparing these two oxygen therapy methods in endoscopic mucosal debridement (31), which also reported similar hypercapnia incidence between the two groups. One study by Sawase et al. (13) defined severe hypercapnia as a partial pressure of carbon dioxide (PaCO_2_) of ≥55 mmHg, whereas the other two studies measured percutaneous partial pressure of carbon dioxide (PtCO_2_) using a PtCO_2_ measuring device, with a mean PtCO_2_ of ≥20 mmHg, which has been previously validated (33). Despite excluding these studies with different hypercapnia measurement criteria, the analysis still did not yield statistically significant results. Most studies investigating HFNC in upper gastrointestinal endoscopy have shown no significant difference in carbon dioxide levels between the HFNC group and the COT group at the end of the procedure (21, 34); this result is consistent with the findings of this study. Furthermore, the different oxygen therapy methods employed in the two groups did not significantly affect the duration of the procedure. For example, in one study (21), the procedure time in the HFNC group was only approximately 30 s longer than that in the COT group, with both groups averaging approximately 5 min. However, in another study (23), the procedure time in the HFNC group was significantly shorter than that in the COT group. This contradictory result may be influenced by various factors such as sample size, study design, type of surgery, and interventions. Subgroup analysis was conducted to explore this discrepancy; however, the result did not show statistically significant differences in procedure time between the examination modalities using HFNC and COT. Therefore, additional large-scale, multicenter, and well-designed studies are required to confirm these results and further investigate potential influencing factors.

It is crucial to acknowledge the limitations of this study when interpreting the conclusions. First, the small sample size of the 12 included literature sources may significantly impact the statistical power and introduce discrepancies in the results. Moreover, certain included studies lacked detailed information regarding allocation schemes and blinding methods, which may introduce a risk of bias. Furthermore, variations in the flow rate, device used, and type of sedation utilized in HFNC across different studies can impede the generalizability of the findings to clinical practice. Finally, the inclusion of studies from different countries with significant heterogeneity in the study population may impact the efficacy of the analysis and limit the generalizability of the results. These limitations should be considered when interpreting the findings of this study, and future research should aim to address these issues to provide more robust and applicable evidence on the use of HFNC in gastrointestinal endoscopy.

## 5 Conclusion

In summary, the application of HFNC in gastrointestinal endoscopy has significantly reduced the incidence of hypoxemia and the need for airway interventions compared to when COT is used. HFNC effectively enhances oxygenation, improves SpO_2_ levels, and reduces the requirement for airway interventions, thereby contributing to improved patient safety and procedural continuity. However, this study has certain limitations that should be acknowledged. Therefore, more scientifically rigorous, large-scale, multicenter, and high-quality RCTs are required to provide further evidence on the effectiveness and safety of HFNC in patients undergoing gastrointestinal endoscopy.

## Data availability statement

The original contributions presented in the study are included in the article/supplementary material, further inquiries can be directed to the corresponding authors.

## Author contributions

CW: Writing – original draft, Writing – review & editing. SM: Writing – review & editing, Writing – original draft. LJ: Writing – original draft. JW: Writing – original draft. LY: Writing – original draft, Writing – review & editing. YW: Writing – original draft, Methodology, Writing – review & editing.
